# Cranioplasty following ventriculoperitoneal shunting: lessons learned

**DOI:** 10.1007/s00701-020-04597-y

**Published:** 2020-10-03

**Authors:** Dorian Hirschmann, Beate Kranawetter, Constanze Kirchschlager, Matthias Tomschik, Jonathan Wais, Fabian Winter, Matthias Millesi, Johannes Herta, Karl Roessler, Christian Dorfer

**Affiliations:** Department of Neurosurgery, Medical University Vienna, Spitalgasse 23, 1090 Vienna, Austria

**Keywords:** Cranioplasty, Complications, Shunt, Epidural hemorrhage, Fluid collection

## Abstract

**Objective:**

Cranioplasty (CP) is considered as a straightforward and technically unchallenging operation; however, complication rates are high reaching up to 56%. Presence of a ventriculoperitoneal shunt (VPS) and timing of CP are reported risk factors for complications. Pressure gradients and scarring at the site of the cranial defect seem to be critical in this context. The authors present their experiences and lessons learned.

**Methods:**

A consecutive series of all patients who underwent CP at the authors’ institution between 2002 and 2017 were included in this retrospective analysis. Complications were defined as all events that required reoperation. Logistic regression analysis and chi-squared test were conducted to evaluate the complication rates according to suspected risk factors.

**Results:**

A total of 302 patients underwent cranioplasty between 2002 and 2017. The overall complication rate was 17.5%. Complications included epi-/subdural fluid collection (7.3%) including hemorrhage (4.6%) and hygroma (2.6%), bone graft resorption (5.3%), bone graft infection (2.0%), and hydrocephalus (5.7%). Overall, 57 patients (18.9%) had undergone shunt implantation prior to CP. The incidence of epi-/subdural fluid collection was 19.3% in patients with VPS and 4.5% in patients without VPS, OR 5.1 (95% CI 2.1–12.4). Incidence of hygroma was higher in patients who underwent early CP. Patients with temporary shunt ligation for CP did not suffer from complications.

**Conclusion:**

CP in patients with a VPS remains a high-risk procedure. Any effort to understand the pressure dynamics and to reduce factors that may trigger the formation of a large epidural space must be undertaken.

## Introduction

Decompressive hemicraniectomy (DHC) is a well-established procedure for treatment of patients with elevated intracranial pressure. Underlying pathologies and indications for DHC are diverse; hence, cranioplasty procedures represent a frequently required neurosurgical procedure in a heterogeneous group of patients [1–3]. The procedure itself is often regarded as straightforward and technically unchallenging and is commonly performed by junior residents. However, the rate of associated complications is much higher than encountered in many other neurosurgical procedures and must not be neglected or underestimated [4–7]. These complications include epidural and subdural fluid collection, infection and/or resorption of the bone graft, new-onset seizures, and hydrocephalus [4, 6, 7]. According to previous reports, complication rates after cranioplasty seem to depend on the timing of cranioplasty [8]. In a large series of 754 patients, performance of cranioplasty between 15 and 30 days after initial craniectomy was associated with lower rates of infection, seizures, and bone flap resorption, whereas intervals of > 90 days decreased the hydrocephalus rate and increased the risk of seizures [6]. While presence of a ventriculoperitoneal shunt (VPS) is a reported risk factor for complications in cranioplasty, there is equipoise in the current literature regarding the optimal timing of VPS implantation and cranioplasty in patients undergoing both procedures. Some authors argue that simultaneous performance of VPS implantation and cranioplasty bears higher risks for complications than staged surgery. Contrarily, others reported lower complication rates when these two procedures are performed simultaneously [9–12].

The overarching problem in patients with disturbances of cerebrospinal fluid circulation and a skull bone defect is that the underlying pathology and mechanism of the CSF disturbance is heterogenous and not always explainable according to the common understanding of hydrocephalus. Hence, negative and positive pressures as well as pressure gradients may be present at the time of cranioplasty and changes of the intracranial pressure by these two procedures need to be considered when planning surgery. The impact of programmable valves, the concept of a temporary ligation of the shunt, and stringent rules for patient positioning after cranioplasty in the presence of a VPS have not been discussed in the literature so far [7, 8].

The objective of this study is to present our single-center experience in this complex patient population. We evaluated the effect of timing of cranioplasty and presence of a VP shunt on the incidence of post-cranioplasty complications with special attention to nuances in the perioperative management of these patients that may have an influence on the complication rate.

## Methods

The authors retrospectively evaluated a consecutive series of patients who underwent cranioplasty between 2002 and 2017 at their institution with emphasis on the postoperative complication rate. The following data were extracted from the electronic medical records: Underlying pathology and indication for DHC, timing of cranioplasty and VPS implantation, and details on perioperative management and postoperative complications. Complications were defined as any requiring reoperation and included epidural and subdural fluid collections, hydrocephalus, bone graft infection, and bone graft resorption. Epi- and subdural fluid collections were further divided into hemorrhage and hygroma. Preoperative CT images prior to cranioplasty were reviewed and the status of the cranial defect (i.e., the level of the brain in relation to the assumed skull line) was documented.

The operative techniques of DHC and cranioplasty were performed as described previously [13]. Autologous bone flaps were stored after DHC for cranioplasty. In cases of microbiological contamination or irreparable damage, artificial implants were used. According to the surgeon’s preference, bone cement (PALACOS®) or a custom-made PEEK implant were used for repair of the cranial defect. Our institutional policy for the management of hydrocephalus in this patient population over the study period was as follows: If ventricular enlargement with a bulging brain was encountered after the acute phase, a VP shunt with a programmable valve was implanted on the opposite side of the cranial defect. If the clinical and radiological information were not clear enough to diagnose hydrocephalus and to decide whether a VP shunt was needed or not, a diagnostic lumbar puncture to withdraw about 30 ml was performed. The initial adjustment of the valve was set to 200 mm H_2_O to avoid a sunken brain. Depending on the status of the cranial defect over time, the valve was then adjusted appropriately. Similarly, attention was given to the positioning of the patient in order to better understand the pressure dynamics before the cranioplasty procedure was planned. If a valve setting of 200 mm H_2_O (CODMAN®HAKIM® Programmable Valve) and the order to keep the patient’s upper body as flat as possible were insufficient to prevent a sunken brain, the cranioplasty was performed with prior clip ligation of the shunt through a retroauricular incision. This ligation was kept as long as deemed acceptable with regard to the size of the ventricles and the clinical status of the patient. This maneuver was hypothesized to prevent epi-/subdural fluid collections underneath the cranioplasty and to enable sufficient attachment of the dural scar layer to the bone. Furthermore, a gravitational valve (MIETHKE®) was added to the system through a supraclavicular incision in mobile patients.

### Statistical analysis

We divided patients into two groups for the analysis, i.e., patients with and without a VPS at the time of cranioplasty. Complication rates were calculated using Fisher’s exact test. The same method was used for comparison between one-stage surgery and two-stage surgery in patients who underwent VPS implantation prior to cranioplasty. To evaluate the impact of the preoperative VPS valve setting as well as timing of cranioplasty on the incidence of complications, logistic regression analysis for the continuous variables was applied. Furthermore, a categorical variable was created, dividing patients into groups according to timing of cranioplasty as reported previously [6, 9]. Relevant risk factors in these groups were compared using chi-squared test and odds ratios were calculated. *P* values < 0.05 were considered significant. Statistical analysis was performed with IBM SPSS Statistics 24. This study has been approved by the ethics committee of the authors’ institution (EK 2244/2017).

## Results

Overall, a total number of 302 patients underwent cranioplasty. The median age at cranioplasty was 48 years (1–80 years) and 160 patients (53.0%) were male. In 283 patients (93.7%), an autologous bone graft was used for cranioplasty and a synthetic graft in 19 patients (6.3%). For synthetic reconstruction, a PEEK implant was used in twelve patients (4.0%) and bone cement in seven patients (2.3%). As listed in Table 1, indications for craniectomy were evenly distributed including ischemic stroke, traumatic brain injury, subarachnoid hemorrhage, and intraparenchymal hemorrhage. Only a minority of procedures was performed for other indications including brain abscess, tumor, and fulminant encephalitis with edema. Craniectomy was performed on the left in 39.4% (119/302), on the right in 56.6%, and bilaterally in 4.0% (12/302). The overall median follow-up was 33.0 months (0.0–211.0 months). In 88.4% of patients, a minimum follow-up of 1 month was available.

**Table 1:** Indications for craniectomy. An even distribution of indications for craniectomy among the cohort of 302 patients who underwent cranioplasty

Indication for craniectomy	(*n* = 302)
Subarachnoid hemorrhage	86 (28.5%)
Ischemic stroke	77 (25.5%)
Intraparenchymal hemorrhage	66 (21.9%)
Traumatic brain injury	66 (21.8%)
Other	7 (2.3%)

None of the patients died due to immediate surgery-related complications. One patient died of pulmonary embolism 1 week after cranioplasty. The overall complication rate was 17.5%. A complete overview of complication rates is given in Table 2. The median time between craniectomy and cranioplasty was 92.5 days (8–2926 days). Patients were grouped according to timing of cranioplasty as follows: ≤ 30 days, 31 to 60 days, 61 to 90 days, and > 90 days after craniectomy. The number of patients assigned to each group and corresponding complication rates are given in Table 3.

**Table 2:** Complications in patients with and without a shunt at cranioplasty. The incidence of complications after cranioplasty is dependent on the presence of a shunt during the operation. Incidence of epi-/subdural fluid collection was higher in patients who underwent shunt implantation before cranioplasty

Complication	W/o shunt (*n* = 245)	Shunt (*n* = 57)	Total (*n* = 302)	*P* value (chi^2^/Fisher’s exact)
Epi-/subdural fluid collection	11 (4.5%)	11 (19.3%)	22 (7.3%)	0.001
Hemorrhage	7 (2.9%)	7 (12.3%)	14 (4.6%)	0.007
Hygroma	4 (1.6%)	4 (7.0%)	8 (2.6%)	0.044
Bone graft resorption	13 (5.3%)	3 (5.3%)	16 (5.3%)	1.000
Infection	5 (2.0%)	1 (1.8%)	6 (2.0%)	1.000
Hydrocephalus	14 (5.7%)	-	14 (4.6%)	-
Overall patients with complications	39 (15.9%)	14 (24.6%)	53 (17.5%)	0.126

**Table 3:** Complications according to timing of cranioplasty. Patients were divided into groups according to time between craniectomy and cranioplasty. Complications of each group are shown, hygroma being the only complication associated with time of cranioplasty. *P* values were calculated with chi-squared and Fisher’s exact test

Complication	< 30 days (*n* = 44)	30–60 days (*n* = 32)	61–90 days (*n* = 73)	> 90 days (*n* = 153)	Total (*n* = 302)	*P* value
Epi-/subdural fluid collection	6 (13.6%)	3 (9.4%)	6 (8.2%)	7 (4.6%)	22 (7.3%)	0.204
Hemorrhage	1 (2.3%)	1 (3.1%)	6 (8.2%)	6 (3.9%)	14 (4.6%)	0.389
Hygroma	5 (11.4%)	2 (6.3%)	0 (0.0%)	1 (0.7%)	8 (2.6%)	< 0.001
Bone graft resorption	0 (0.0%)	2 (6.3%)	4 (5.5%)	10 (6.5%)	16 (5.3%)	0.393
Infection	1 (2.3%)	0 (0.0%)	2 (2.7%)	3 (2.0%)	6 (2.0%)	0.830
Hydrocephalus	5 (11.4%)	2 (6.3%)	3 (4.1%)	4 (2.6%)	14 (4.6%)	0.104
Overall patients with complications	11 (25.0%)	6 (18.8%)	14 (19.2%)	22 (14.4%)	53 (17.5%)	0.405

### Shunt-dependent hydrocephalus and associated complications

In 57 patients (18.9%), a VPS was present at time of cranioplasty and 14 patients (4.6%) required shunt implantation due to hydrocephalus after cranioplasty. There was a trend for a higher incidence of post-cranioplasty hydrocephalus in patients who underwent early cranioplasty; however, this was not a significant finding (Table 3).

Overall, 22 of 302 patients (7.3%) required reoperation due to epi- or subdural fluid collection including epidural hemorrhage and epi-/subdural hygroma. The incidence of this complication was significantly higher in patients with a shunt present at time of cranioplasty. This association was still significant when analyzed separately for epidural hemorrhage and epi-/subdural hygroma (Table 2). The odds ratios were 4.8 (95% CI 1.6–14.2) for epidural hemorrhage, 4.6 (95% CI 1.1–18.8) for subdural hygroma, and 5.1 (95% CI 2.1–12.4) for the combined outcome. Of note, complication rates did not differ between patients who underwent one-stage VPS implantation prior to cranioplasty (*n* = 14) and patients undergoing two-stage VPS implantation and cranioplasty (*n* = 43). Neither did the preoperative setting of the VPS valve influence the rate of complications.

The rate of overall epi-/subdural fluid collection was independent of the timing of cranioplasty; however, the incidence of hygroma was significantly higher in patients who underwent cranioplasty < 30 days and 30–60 days after craniectomy. Logistic regression showed borderline significance (*P* = 0.058) of the continuous variable.

There were four patients who underwent temporary ligation of the VP shunt according to the above stated policy of our institution. Ligation of the shunt was performed simultaneously with cranioplasty in two cases and 3 days/10 days prior to cranioplasty in two other cases. Shunt ligation was maintained for 1 to 13 days after cranioplasty. None of these patients suffered from epi- or subdural fluid collection after cranioplasty.

In 9 patients, a gravitational shunt valve was implanted prior to cranioplasty, one of whom suffered from epidural hemorrhage after CP. Implantation of a gravitational valve prior to cranioplasty was not associated with any changes in complication rates.

The radiological analysis of cranial defects showed that epi- or subdural fluid collection in patients with VPS occurred only if the CT scan prior to cranioplasty showed a sunken or bulging brain and not if the brain was at the level of the assumed skull line.

### Infection and bone graft resorption

In 6 patients (2.0%), explantation of the bone graft had to be performed due to infection. Furthermore, 16 (5.3%) patients underwent repeat cranioplasty after resorption of the initial bone graft. Neither of these complications was dependent on timing of cranioplasty or presence of a VPS at time of cranioplasty.

## Discussion

Shunt-dependent hydrocephalus in patients requiring cranioplasty represents a major challenge in the management of these patients with complication rates up to 56% [6, 10]. Our experience shows that the rate of complications may be reduced if (i) any attempt is made to reduce the size of the artificial epidural space underneath the bone flap; (ii) the cranial defect is at the level of the assumed skull line before CP; (iii) special attention is given to understand the individual patient fluid and pressure dynamics before CP; and (iv) the pre-/postoperative management is individualized to this understanding by measures including the adjustment of the programmable valves, patient positioning, implantation of a gravitational valve, and temporary ligation of the shunt, if needed.

Our results add valuable information to solve the current equipoise regarding the management and timing of cranioplasty in the presence of a hydrocephalus requiring shunt treatment [10, 14, 15]. While there seems to be a consensus among most studies that a VPS is a major risk factor for complications in cranioplasty procedures including subdural or epidural fluid collections, intracerebral hemorrhage, infections, bone flap resorption, and seizures, the timing of these two procedures, i.e., as a one-stage or two-stage procedures, is less clear. Two case series argued that staged surgery is associated with fewer postoperative complications [10, 11]. Schuss et al. investigated 41 cranioplasty procedures with simultaneous VPS placement in 17 patients (41%) and staged VPS placement in 24 patients. In 21 out of the 24 staged operations, the VPS placement was done prior to CP. Overall, simultaneous procedures in this series were associated with a significantly higher rate of postoperative complications compared with staged procedures (47% vs. 12%; *P* = 0.03, OR 6.2, 95% CI 1.33–29.0) [11]. Accordingly, Heo et al. reviewed 51 patients who had undergone CP and VPS placement of which 19 were performed at different time points and 32 at the same time. Again, the complication rate in simultaneous procedures exceeded the one in staged procedure reaching 56% and 21%, respectively [10]. In contrast to these findings, Meyer et al. reported in their series of 58 patients who underwent CP and VPS in simultaneous or staged procedures no difference in the rate of complications [12]. In our study, there was no difference in complication rates between patients undergoing one-stage and those undergoing two-stage surgery.

One explanation of these contradicting results may be simply given by a low case number of simultaneously performed surgeries in our cohort (14 of 57 patients) and the fact that Meyer et al. focused on variables including bone graft infection, VPS infection, and VPS obstruction omitting other complications such as hemorrhage and fluid collections. These latter complications, however, are specifically attributable to the pressure and fluid dynamics in these patients. Conversely, there may be other factors more important than just the question if a simultaneous or separate procedure leads to a lower complication rate. One of the key steps in CP is dissecting the subcutaneous tissue and dura. In patients with CSF disturbances, requiring VPS implantation two factors may complicate and increase the risk for a postoperative complication. First, if CP is performed too early after DHC before scarring has created a sufficiently dense dural scar layer, dissecting the correct interlayer to the subcutaneous tissue plane is challenging and an opening of the CSF space is often unavoidable. This artificial communication to the epidural space may trigger postoperative fluid collections in patients with CSF circulation disturbances. Furthermore, sufficient application of tack-up sutures to the bone graft is not easily feasible in the absence of an intact dural scar layer. This again triggers the creation of a large artificial epidural space underneath the bone flap. This hypothesis is supported by our finding that the incidence of postoperative sub- and epidural hygroma was significantly higher in patients undergoing early cranioplasty (Table 3).

Second, patients with VPS are more likely to develop a sunken skin flap. This increases the difficulty in exposing the dural scar layer and demands the surgeon to lift the scalp with a greater effort. This manipulation may cause repeat contusion and stretching of the subdural content increasing the risk of subdural hemorrhage in these patients. Furthermore, this vulnerable condition is often aggravated by formation of a large epidural space especially in the frontal region of the defect. The application of numerous tack-up sutures in this region is usually insufficient to solve the problem. To overcome these difficulties, we suggest that the cranial defect should be at the assumed skull line and this aim could be achieved by the following measures: (i) the use of a programmable valve allows to adjust the amount of CSF drainage to a level that reduces the degree of a sunken and flaccid cranial defect; (ii) if the cranial defect remains sunken and flaccid despite the highest setting of 200 mm H_2_O, attention should be given to position the upper body of the patient as flat as possible; (iii) if both maneuvers are insufficient to achieve a cranial defect that is at the level of the assumed skull line, a temporary ligation of the shunt should be considered. In our series, this maneuver was necessary in four patients none of whom experienced any complication after CP.

All of these measures aim at reducing the size of the artificial epidural space underneath the bone flap, which is triggered by the pressure gradient caused by the VPS. This might be an even larger concern in simultaneous CP and VPS procedures with high positive pressures indicated by brain bulging above the assumed skull line. If a VPS and CP are performed simultaneously, a rapid pressure gradient change may occur provoking an accumulation of blood and fluids underneath the bone flap epidurally and subdurally. Heo et al. showed in their series that 18 out 32 patients (56%), who had a tense convex cranial defect at the time of a simultaneous procedure, experienced a complication [10]. Accordingly, our analysis of preoperative CT scans showed that the complication rate is significantly reduced if the cranial defect was at the assumed skull line before CP. The fact that the Heo et al. chose a mean shunt pressure setting of 110 mm H_2_O might have contributed to this high rate of complications, although details on the exact valve adjustments in these cases is not given in their report. In our series, no association between valve setting and complication rates could be observed.

### Limitations

Our study is limited due to its retrospective character. The presented maneuvers to reduce the complication rate after CP was only part of the institutional policy and matured over the time course by the lessons learned from this series. Hence, a dedicated protocol would be necessary to confirm our results. However, our experience and lessons learned may guide other neurosurgeons in the difficult task to reduce the high complication rate of this commonly practiced procedure.

## Conclusion

Cranioplasty in patients with shunt-dependent hydrocephalus remains a high-risk procedure and should not be underestimated. Any attempt to understand the pressure and fluid dynamics in this complex patient population should be done. All factors that might trigger the formation of a large artificial space underneath the bone flap should be considered and reduced.
